# Exploration of the Role of m^6^ A RNA Methylation Regulators in Malignant Progression and Clinical Prognosis of Ovarian Cancer

**DOI:** 10.3389/fgene.2021.650554

**Published:** 2021-06-03

**Authors:** Qinglv Wei, Dan Yang, Xiaoyi Liu, Hongyan Zhao, Yu Yang, Jing Xu, Tao Liu, Ping Yi

**Affiliations:** Department of Obstetrics and Gynecology, The Third Affiliated Hospital of Chongqing Medical University, Chongqing, China

**Keywords:** ovarian cancer, m^6^A, TCGA, TME, prognosis, LASSO Cox regression, consensus clustering

## Abstract

Ovarian cancer is the most deadly gynecologic malignancy worldwide and it is warranted to dissect the critical gene regulatory network in ovarian cancer. N6-methyladenosine (m^6^A) RNA methylation, as the most prevalent RNA modification, is orchestrated by the m^6^A RNA methylation regulators and has been implicated in malignant progression of various cancers. In this study, we investigated the genetic landscape and expression profile of the m^6^A RNA methylation regulators in ovarian cancer and found that several m^6^A RNA methylation regulators were frequently amplified and up-regulated in ovarian cancer. Utilizing consensus cluster analysis, we stratified ovarian cancer samples into four clusters with distinct m^6^A methylation patterns and patients in these subgroups displayed the different clinical outcomes. Moreover, multivariate Cox proportional hazard model was used to screen the key m^6^A regulators associated with the prognosis of ovarian cancer and the last absolute shrinkage and selection operator (LASSO) Cox regression was used to construct the gene signature for prognosis prediction. The survival analysis exhibited the risk-gene signature could be used as independent prognostic markers for ovarian cancer. In conclusion, m^6^A RNA methylation regulators are associated with the malignant progression of ovarian cancer and could be a potential in prognostic prediction for ovarian cancer.

## Introduction

Ovarian cancer is a gynecologic malignancy with the most deaths worldwide (Narod, 2016). Nearly 90% of all ovarian cancer is epithelial ovarian cancer, which contributes to 70% of ovarian cancer deaths (Jayson et al., 2014). Despite large efforts toward better prognosis of ovarian cancer patients, the 5-year overall survival rate is lower than 40% because of the high relapse rate and drug resistance (Doherty et al., 2017; Reid et al., 2017). Thus, ovarian cancer remains a threat to women and enhancing understanding of underlying mechanisms and identification of critical gene regulatory network in ovarian cancer progression are conducive to prediction and developing new therapies for ovarian cancer.

m^6^A RNA methylation is the most abundant RNA epigenetic modification in mammals (Roundtree et al., 2017). m^6^A RNA methylation is a reversible and dynamic process mediated by methylation transferases and demethylation transferases (Jia et al., 2011; Zheng et al., 2013; Liu et al., 2014). To date, the complex including methyltransferase-like 3/14 (METTL3/14), and Wilms’ tumor 1-associating protein (WTAP) was identified as the core m^6^A methylation transferases and acts as m^6^A methylation “writers” (Schöller et al., 2018). Other factors including Vir like m^6^A methyltransferase associated (VIRMA, also named as KIAA1429), RNA binding motif protein 15/15B (RBM15/15B), and zinc finger CCCH domain-containing protein 13 (ZC3H13) were also demonstrated to be involved in m^6^A methylation deposition (Schwartz et al., 2014; Meyer and Jaffrey, 2017; Wen et al., 2018). Regarding demethylation transferases, obesity-associated protein (FTO) and alkB homolog 5 (ALKBH5) act as m^6^A methylation “erasers” and could decrease the m^6^A modification in RNA (Jia et al., 2011; Zheng et al., 2013). Besides m^6^A methylation “writers” and “erasers,” a group of RNA binding proteins were found to specifically recognize m^6^A modified RNAs and decide the fate of RNA through regulating RNA splicing, turnover, export and translation (Haussmann et al., 2016; Patil et al., 2018; Zhao et al., 2018). m^6^A methylation “readers” are consisted of YTH domain family YTHDF1-3, YTHDC1-2, insulin-like growth factor 2 mRNA-binding proteins IGF2BP1-3, heterogeneous nuclear ribonucleoprotein A2B1 (HNRNPA2B1), heterogeneous nuclear ribonucleoprotein C (HNRNPC) and embryonic lethal abnormal vision Drosophila like 1 (ELAVL1) (Wang et al., 2014, 2015; Wojtas et al., 2017; Sun et al., 2019). m^6^A RNA methylation is an important RNA epigenetic regulation mode and leads to a complex gene regulatory network through the posttranscriptional control. Recent studies revealed that dysregulation of m^6^A RNA methylation has been significantly implicated in various diseases especially in development of cancers (Geula et al., 2015; Yoon et al., 2017; Chen et al., 2019; Esteve-Puig et al., 2020). For example, endometrial cancer is associated with a reduced level of m^6^A mRNA methylation because of decreased expression of METTL3 and METTL14 and reduced m^6^A methylation promotes the proliferation of endometrial cancer cell (Liu et al., 2018). METTL3 has been also reported to inhibit myeloid differentiation of normal hematopoietic and leukemia cells (Vu et al., 2017). On the contrary, METTL3 was up-regulated in human hepatocellular carcinoma and lung cancer (Chen et al., 2018; Choe et al., 2018). METTL3 directed m^6^A modification of tumor suppressor gene SOCS2 and silenced its expression depending on YTHDF2-mediated degradation pathway, which promoted the progression of hepatocellular cancer (Chen et al., 2018). In lung cancer, METTL3 enhanced circularization and translation of m^6^A modified mRNAs, and thus promoted oncogenesis (Choe et al., 2018). FTO was revealed as the first RNA m^6^A demethylase which is highly expressed in several AML subtypes (Barbieri et al., 2017). FTO plays an oncogenic role through facilitating cell proliferation and leukemogenesis, and inhibiting all-trans-retinoic acid-mediated differentiation of leukemia cells. R-2HG, a specific small molecule inhibitor of FTO, displays anti-leukemia activity by suppressing FTO/m^6^A/MYC signaling (Su et al., 2018). In our previous study, we found that the m^6^A “reader” YTHDF1 aggravated ovarian cancer progression by enhancing EIF3C translation in an m^6^A-dependent manner (Liu et al., 2020). However, the role of m^6^A RNA methylation-mediated gene regulatory network in diagnosis and treatment of ovarian cancer is largely unexplored.

In this study, we examined the genetic variations and gene expression of m^6^A RNA methylation regulators. We found several m^6^A RNA methylation regulators were amplified and up-regulated in ovarian cancer. We also identified the hub genes by protein interaction analysis and a signature gene for prognostic predication of ovarian cancer. Moreover, we classified the ovarian cancer patients into four subgroups with distinct overall survivals based on the expression of 20 m^6^A RNA methylation regulators. Our study demonstrated that m^6^A RNA methylation regulators have an important value in prognostic prediction for ovarian cancer.

## Materials and Methods

### m^6^A Regulators

According to the mRNA expression detected by the Cancer Genome Atlas (TCGA) database, 20 m^6^A regulators including 7 “writers” (KIAA1429, METTL3, METTL14, RBM15, RBM15B, WTAP, ZC3H13), 2 “erasers” (ALKBH5, FTO) and 11 “readers” (YTHDC1-2, YTHDF1-3, IGF2BP1-3, HNRNPA2B1, HNRNPC, ELAVL1) were analyzed in this study.

### m^6^A Regulators Mutation and Copy Number Variation Analysis

The workflow of our study was shown in Supplementary Figure 1. DNA mutation and copy number variation data were downloaded from the Genomic Data Commons (GDC)^1^. The R bioconductor package maftools was used for somatic mutation investigation of the m^6^A regulators (Mayakonda et al., 2018). The Pan cancer project of TCGA-OV was used in our study.

### Microarray Datasets and Differentially Expressed Genes Analysis

Gene Expression Omnibus (Choe et al., 2018) database was selected to study the differential expression of 20 m^6^A regulators between normal tissues and tumor tissues. Datasets containing 4 normal samples with expression of all the 20 m^6^A regulators were selected in this study. 4 normalized matrix files (GSE27651, GSE52037, GSE54388, and GSE66957) were selected and downloaded from GEO. Batch effects were corrected by sva package (Leek and Storey, 2008) and differential expression was calculated by limma package (Ritchie et al., 2015).

### Interaction Between 20 m^6^A Regulators

Protein-Proterin interaction was constructed using the STRING 11.0b website^2^. The RNA expression correlation among the m^6^A regulators was conducted by R package corrplot. RNA expression data (FPKM) was downloaded from GDC.

### Clustering Analysis of 20 m^6^A Regulators

ConsensusClusterPlus package (Wilkerson and Hayes, 2010) was used to classify the TCGA patients to identify distinct m^6^A phenotype based on the expression of 20 m^6^A regulators and 1,000 times repetitions were conducted to make sure our classification is stable.

### Tumor Microenvironment Cell Infiltration Estimate

The TCGA ovarian cancer immune cell type information predicted by deconvolution algorithm was downloaded from the CIBERSORT website^3^. ESTIMATE was conducted by estimate package to quantify the overall stromal cells, immune cells, and tumor purity of individual TCGA patients. To further qualify the relative levels of different activated or naïve immune cell types infiltration among the distinct m^6^A clusters, the enrichment score of 23 immune cell types which reported in pancancer (Charoentong et al., 2017) were calculated by ssGSEA (single-sample gene-set enrichment analysis) algorithm from gsva package (Hänzelmann et al., 2013).

### Cluster Function Annotation

First of all, R package GSVA was used to study the KEGG pathway enrichment among different m^6^A clusters. Secondly, functional annotation of each m^6^A clusters was performed by R package ClusterProfiler (Yu et al., 2012) among the top 500 expressed genes. Thirdly, differential gene expression analysis was conducted using limma package between each cluster and the rest patients, and then used overexpressed analysis by ClusterProfiler package to identify the individual cluster.

### Survival Analysis

Overall survival analysis was conducted using the integrated microarray datasets^4^ through Kaplan-Meier method. Survival and survminer packages (Scrucca et al., 2007) were used to establish the univariate Cox proportional hazards model and overall survival plot. Genes with the p < 0.1 were selected to lasso regression. Receiver operating characteristic curve (ROC) and area under curve (AUC) were calculated by R package survivalROC (Heagetry and Zheng, 2005). Patients with survival information were randomly divided into two subgroups (75% in training group and 25% in test group) by createDataPartition function from caret package. Four gene risk signature and their corresponding coefficient were determined in the training group by glmnet function. Risk score was calculated for each patient using prediction function. The best cutoff value for our model was selected as follow: true positive (TP) and false positive (FP) of every patient in training group was calculated through survivalROC function, Risk score of the patients with the minimum value of the formula (TP-1)2 + FP2 was determined as the best cutoff value. This cutoff value was used in training group, test group and external validation set to divide the sample into high-risk group and low-risk group. R package forestplot and survminer were used for visualized the Cox results and survival curves, respectively.

### Statistical Analysis

Co-occurrence of CNV and mRNA expression correlation among different m^6^A regulators were calculated by Spearman correlation analyses by corrplot package. Kruskal-Wallis test was employed to compare gene expression among different samples. R 4.0.3 was used for all the statistical analysis in this study. p < 0.05 is the significance threshold for all the data.

## Results

### Landscape of Genetic Variation and Expression Patterns of m^6^A Regulators in Ovarian Cancer

**FIGURE 1:**
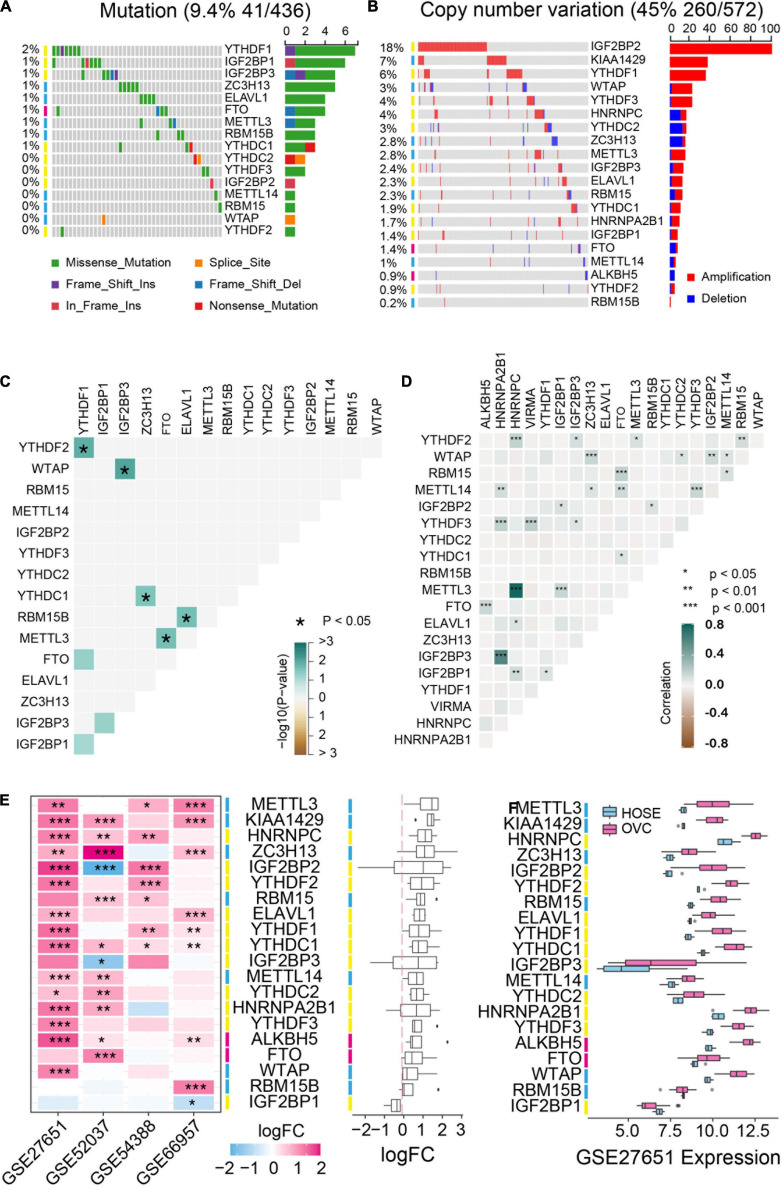
Genetic variation and expression of m^6^A regulators in ovarian cancer. **(A)** The mutation frequency of m^6^A regulators in ovarian cancer available at TCGA database by using maftools package. **(B)** Copy number variations of m^6^A regulators in ovarian cancer available at TCGA database by using cBioPortal (http://cbioportal.org). **(C)** The mutation co-occurrence and exclusion analysis for m^6^A regulators. **(D)** The copy number variation co-occurrence and exclusion analysis for m^6^A regulators. **(E)** The mRNA expression alterations of m^6^A regulators in four independent GEO microarray datasets. **(F)** The mRNA expression of m^6^A regulators in GSE27651 cohort. HOSE, human ovarian surface epithelium, OVC, ovarian cancer. *P < 0.05, **P < 0.01, ***P < 0.001.

We first analyzed the mutation status and copy number variation of 20 m^6^A regulator genes including m^6^A “writers,” “erasers” and “readers” in TCGA ovarian cancer database. These genes displayed different copy number variations in ovarian cancer but low frequency of mutations occurred in all these genes (Figure 1A). *IGF2BP2*, *KIAA1429* and *YTHDF1* genes were highly amplified with amplification frequencies of 18%, 7% and 6%, respectively. *HNRNPC*, *YTHDC2* and *ZC3H13* genes were depleted in ovarian cancer (Figure 1B). Moreover, we analyzed the co-occurrence of DNA mutation and amplification among the m^6^A regulators respectively. Co-occurrence of DNA mutation is rarely and only 5 of 8 pair of genes significantly co-exist in the same patients (Figure 1C). DNA copy number variation is rather pervasive and all the co-exist copy number variation are positive related (Figure 1D). Additionally, we selected four GSE datasets to examine the expression of m^6^A-related genes and found that these genes were usually up-regulated in ovarian cancer compared to normal tissues (Figures 1E,F).

### Interaction and Correlation Analysis Between m^6^A RNA Methylation Regulators

**FIGURE 2:**
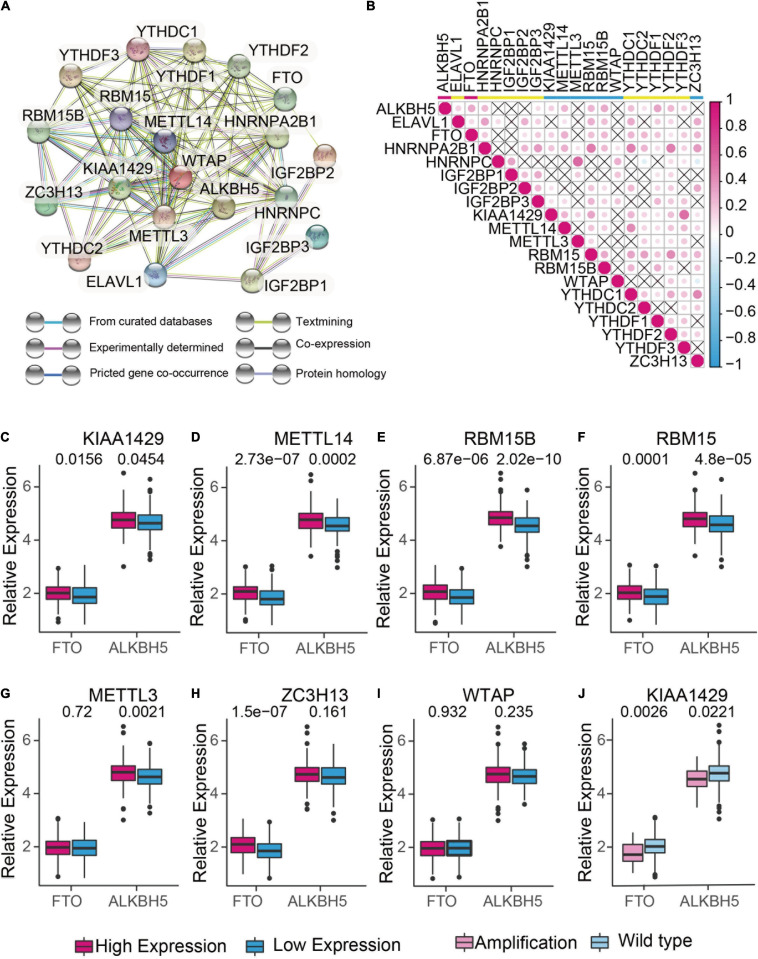
The interaction and correlation analysis between m^6^A regulators in ovarian cancer. **(A)** Protein-Protein Interactions among the 20 m^6^A RNA methylation regulators using STRING 11.b. **(B)** Spearman correlation analysis of 20 m^6^A RNA methylation regulators in TCGA cohorts using R. **(C-I)** Correlation analysis between the expression of “erasers” (FTO and ALKBH5) and “writers” (KIAA1429, METTL14, RBM15, RBM15B, METTL3, ZC3H13, and WTAP). Median expression value was used to divide the patients into high and low expression groups. **(J)** Difference in the gene expression of “erasers” between KIAA1420-amplified and wild types.

To understand the mutual interaction of 20 m^6^A RNA methylation regulators, a protein-protein interaction (PPI) network using Cytoscape was constructed based on the STRING 11.0b database. As shown in Figure 2A, the 20 m^6^A RNA methylation regulators displayed the complex interactions. The writers including METTL14 and WATP interacted with each other and were the hub genes. Moreover, the correlation analysis was conducted to analyze the correlation among these regulators in ovarian cancer. Part of the different m^6^A RNA methylation regulators showed weakly to moderately positive correlation (Figure 2B). Among 20 m^6^A RNA methylation regulators, YTHDF2 was positively correlated with all of the m^6^A RNA methylation regulators except YTHDC2 (Figure 2B). We also found that tumors with a high expression of writer genes (METTL14, RBM5B, RBM15, and KIAA1429) co-expressed with a high expression of “eraser” genes FTO and ALKBH5, whereas a high expression of writer gene WTAP had no correlation with the expression of FTO and ALKBH5 (Figures 2C-I). Considering the high amplification frequency of KIAA1429, we analyzed the differential expression of “eraser” genes in tumors with the distinct copy number variations. We found that both of “eraser” genes were down-regulated in KIAA1429-amplified tumors compared to wide-type tumors (Figure 2J). These results demonstrated that m^6^A RNA methylation regulators formed a complex regulatory network which contributed to the dynamics of m^6^A RNA methylation in ovarian cancer.

### Consensus Clustering of m^6^A RNA Methylation Regulators Identified Four Clusters of Ovarian Cancer With Distinct Clinical Outcomes

**FIGURE 3:**
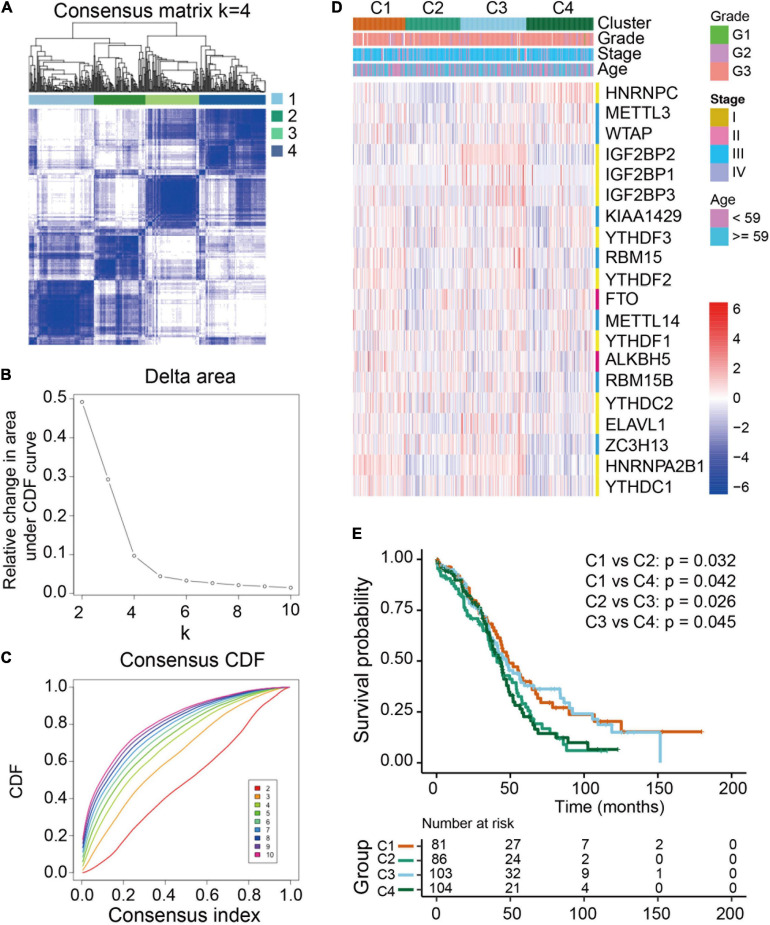
Differential clinicopathological features and overall survival of ovarian cancer in four clusters with distinct m^6^A RNA methylation regulator features. **(A)** Consensus clustering cumulative distribution function (CDF) for *k* = 2-10. **(B)** Relative changes in the area under the CDF curve at k = 2-10. **(C)** Consensus clustering matrix for *k* = 4. **(D)** Heatmap with the clinical and pathological characterizations for clusters according to m^6^A RNA methylation regulator consensus clustering. **(E)** Kaplan-Meier overall survival curves of the four clusters.

To examine the clinical relevance of m^6^A RNA methylation regulators in ovarian cancer, the ConsensusClusterPlus tool was used to separately cluster the TCGA ovarian cancer samples into subgroups according to the gene expression patterns of m^6^A RNA methylation regulators. Four distinct modification patterns (cluster 1-4) were identified using unsupervised clustering while *k* = 4 (Figures 3A-C). Significant differences were found among these four subgroups regarding tumor grade and FIGO stage (Figure 3D). Furthermore, prognostic analysis for the four main m^6^A modification patterns in Figure 3E revealed that cluster 1 (C1) and cluster 3 (C3) had a better overall survival compared to cluster 2 (C2) and cluster 4 (C4).

### Evaluation of TME Cell Infiltration in Distinct m^6^A Modification Patterns

**FIGURE 4:**
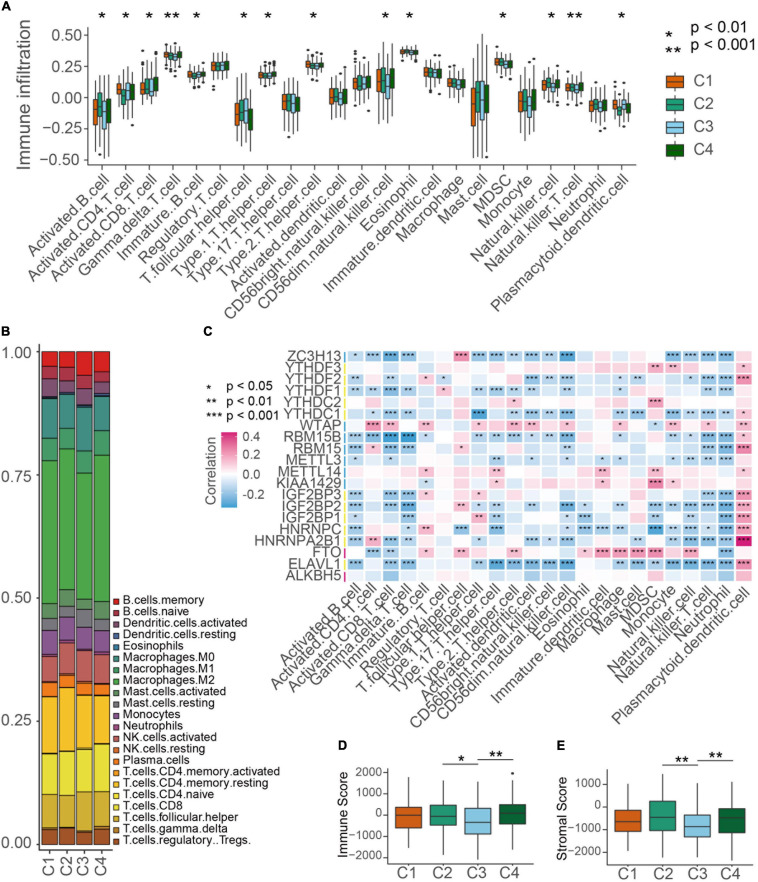
TME cell infiltration characteristics in distinct m^6^A modification patterns. **(A)** The abundance of each TME infiltrating cell type in four m^6^A modification patterns. The TME infiltrating cell types were defined by ssGSEA methods. The upper and lower ends of the boxes represented interquartile range of values. Kruskal-Wallis test was used to calculate the significance between poor prognosis groups and good prognosis groups. *P < 0.01, **P < 0.001. **(B)** The component differences of immune cells among the four m^6^A modification patterns analyzed by CIBERSORT. **(C)** The correlation between each m^6^A regulator mRNA levels and each TME infiltration cell type ssGSEA scores using spearman analyses. Red color means positive correlated and blue color means negative correlated. *P < 0.05, **P < 0.01, ***P < 0.001. **(D,E)** Differences of immuneScore and stromaScore in four subgroups by ESTIMATE. *P < 0.05, **P < 0.01.

Then we evaluated the landscape of TME cells in 4 subgroups with distinct m^6^A modification patterns. As shown in Figures 4A,B, we found 23 TME cells presented different changes in infiltration among subgroups. For most immune cells, the relative quantity of immune cells in the C2 group and the C4 group was significantly higher than that in the C1 group and C3 group (Figure 4B). The correlation between the m^6^A RNA methylation regulators and 23 immune cells was analyzed. We found that the expression levels of most m^6^A RNA methylation regulators were highly related to the abundances of multiple immune cells (Figure 4C). Unexpectedly, activated B cells, eosinophil cells and natural killer cells were significantly enriched in the C2 subgroup, but patients in the C2 group did not present an advantaged prognosis (Figure 4B). Consistently, we used ESTIMATE algorithm to evaluate the immune activity in distinct m^6^A modification subgroups and found that the C2 or C4 subgroups exhibited a higher immune score than the C3 subgroup (Figure 4D). Stroma surrounding tumor cell nests was demonstrated to contribute to the immune excluded phenotype of tumors and thus the stroma activity of m^6^A modification subgroups was evaluated (Chen and Mellman, 2017). Figure 4E showed that the stroma activity in the C2 or C4 subgroups was also higher than that in the C3 subgroup.

### Characteristics of Transcriptome Traits in Clustering Subgroups

**FIGURE 5:**
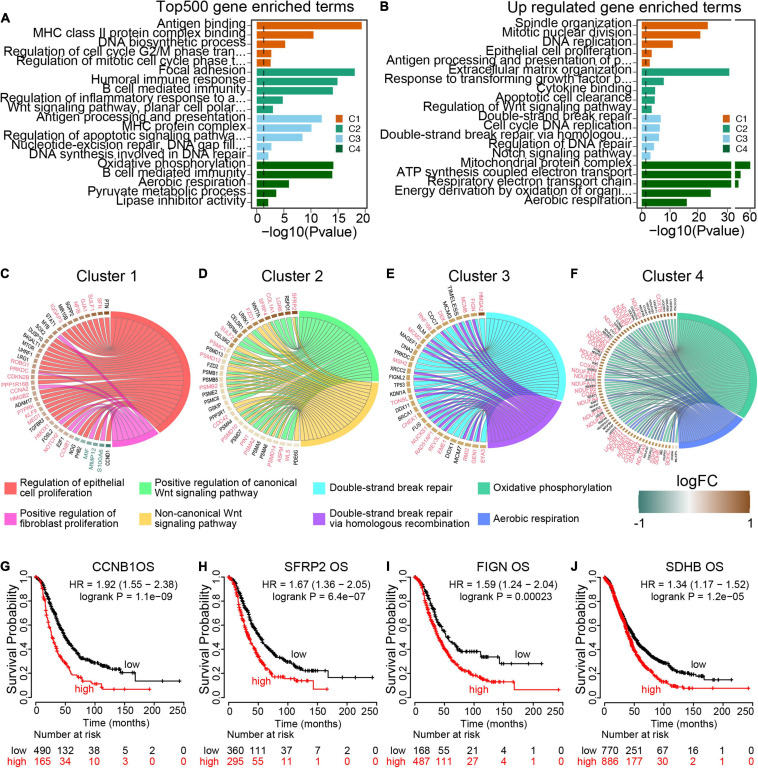
Transcriptome traits in distinct m^6^A modification patterns. **(A)** GO terms enrichment using the top 500 expressed genes in each m^6^A modification patterns. **(B)** GO terms enrichment using the upregulated genes from each cluster compared to the rest cases. **(C–F)** Circos plot exhibits the essential cancer associated GO term clusters of the four m^6^A modification patterns with the differential expressed genes (fold change >1.5 and *p* value <0.05). Genes with good prognosis in high grade serous ovarian cancer are highlighted by red color, and the green for good prognosis genes. Each square represents the fold change of genes between each m^6^A modification patterns and the rest cases. Brown color for upregulated genes and green color for downregulated genes. **(G–J)** Overall survival curves of representative genes enriched in each m^6^A modification patterns.

To further explore the transcriptomic characterization of these m^6^A modification phenotypes, the top 500 expressed genes in each cluster were collected for gene ontology (GO) enrichment analysis using R ClusterProfiler packages. Consistent with the TME immune cell infiltration patterns, all the four m^6^A clusters top 500 expressed genes enriched in immune associated pathways (Figures 5A,B and Supplementary Figure 2A), which demonstrated that m^6^A modification is implicated in ovarian cancer TME. We also determined phenotype-related differential expression genes (DEGs) in different m^6^A clusters and performed GO enrichment analysis. The results showed that cell proliferation associated genes were enriched in Cluster 1 (Figure 5C). And both the canonical and non-canonical Wnt pathways were up-regulated in Cluster 2 (Figure 5D). For Cluster 3, genes involved in DNA repair pathways including homologous recombination and mismatch repair were significant up-regulated (Figure 5E). And energy metabolism pathways such as oxidative phosphorylation and aerobic respiration were highly expressed in Cluster 4 (Figure 5F). Then GSVA was conducted to investigate the potential KEGG pathways mediated by m^6^A regulators. We compared each m^6^A cluster with the other clusters and determined 168 phenotype-associated KEGG pathways with the threshold of *p* < 0.05. As expected, DNA repair pathways were enriched in Cluster 1, and pathways associated with energy metabolism exhibited remarkably high expression in m^6^A Cluster 4 (Supplementary Figure 2B).

To identify target genes regulated by m^6^A regulators, we visualized the typical pathways of the four m^6^A modification phenotype clusters. Of 38 DEGs expressed in Cluster 1 specifically, 20 genes were markedly associated with ovarian cancer overall survival, including 17 up-regulated genes with poor prognosis and 3 down-regulated genes with good prognosis (Figure 5G and Supplementary Figure 2C). All the 35 DEGs within the Wnt pathway were up-regulated and out of 16 (45.71%) genes were oncogenes in ovarian cancer (Figure 5H and Supplementary Figure 2D). In Cluster 3, 32 homologous recombination repair associated genes were up-regulated, of which 16 genes predicted poor prognosis in ovarian cancer (Figure 5I and Supplementary Figure 2E). Among the 72 aerobic respiration related DGEs in Cluster 4, there were 33 (45.83%) genes highly associated with ovarian cancer survival (Figure 5J and Supplementary Figure 2F). All these results demonstrated that the m^6^A regulators were implicated in the prognosis of ovarian cancer, and more importantly, different pathways were activated to regulate tumor progression in different m^6^A modification phenotype clusters with distinct prognosis states.

### Construction and Validation of a Risk Signature With Four Selected m^6^A Methylation Regulators

**FIGURE 6:**
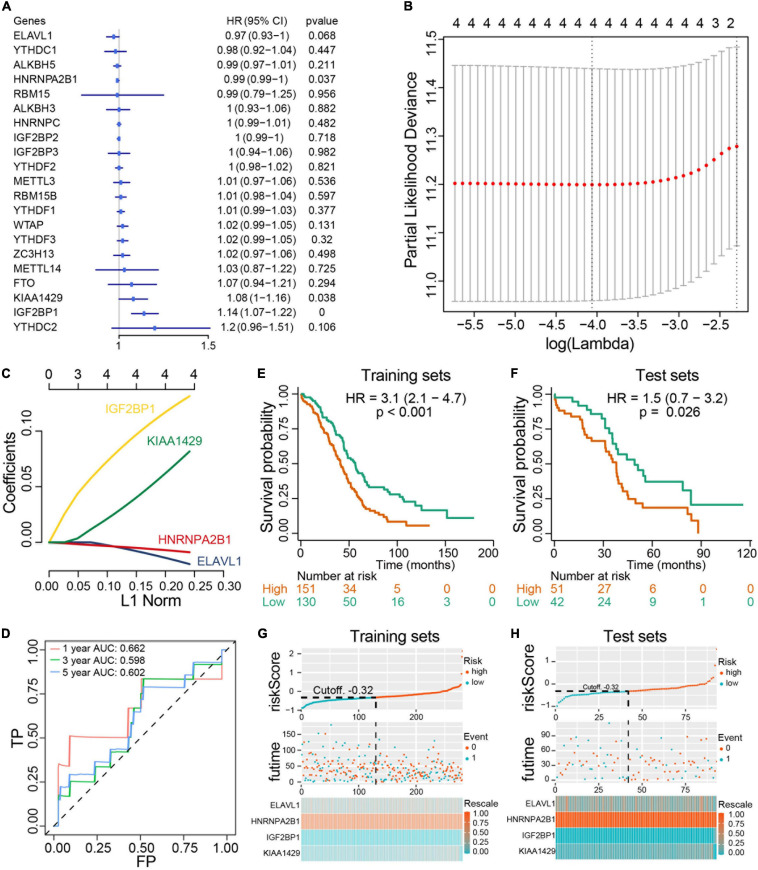
Construction and evaluation of prognostic risk signature with four m^6^A RNA methylation regulators in ovarian cancer cohorts. **(A)** Univariate Cox regression analysis results show the p values and hazard ratios (Liu et al.) with confidence intervals (CI) of the 14 differentially expressed m^6^A RNA methylation regulatory genes. **(B,C)** LASSO Cox regression analysis results show the identification of the 4 prognostic risk signature genes, and the risk score model is: risk score = 0.12218 × IGF2BP1 + 0.0687 × KIAA1429 - 0.0158 × ELAVL1 - 0.00763 × HNRNPA2B1. **(D)** The ROC curve for evaluating the prediction efficiency of the prognostic signature. **(E,F)** The survival analysis of training set and test set. Both two set were divided by the cutoff value according risk score calculated by LASSO multivariate Cox model. **(G,H)** The distributions of prognostic signature-based risk scores and its corresponding expression profiles in the training set and test set. The red dots represent high-risk patients and green dots represent low-risk patients.

We next investigated the prognostic value of m^6^A RNA methylation regulators in ovarian cancer. A univariate Cox regression analysis was performed in training set concerning the expression levels of m^6^A RNA methylation regulators to identify the regulators associated with overall survival in TCGA ovarian cancer cohort. We found that 3 out of 20 regulators were significantly associated with overall survival, among which KIAA1429 and IGF2BP2 belonged to risky genes with HR > 1 while HNRNPA2B1 was the only protective gene with HR < 1 (Figure 6A). To more precisely predict the prognosis of ovarian cancer with m^6^A RNA methylation regulators, we applied the LASSO Cox regression algorithm to develop a risk signature in the training set. According to the minimum criteria, the survival risk score model was established as follow: risk score = −0.0158 ELAVL1 −0.00763 HNRNPA2B1 + 0.12218 IGF2BP1 + 0.0687 KIAA1429 (Figures 6B,C). The ROC curves displayed that prognosis prediction using the risk signature had an area under the ROC curve (AUC) value of 0.662 (1 year), 0.598 (3 years) and 0.602 (5 years) in training set (Figure 6D). To detect the prognostic role of the four-gene risk signature, we divided the ovarian cancer patients in both training set and test set into low-risk and high-risk group based on the lasso cutoff risk score calculated above and compared the overall survival of patients in different subgroups. Results indicated patients in high-risk group exhibited a worse overall survival than low-risk patients in both sets (Figures 6E,F). The distributions of four-gene signature-based risk scores as well as its corresponding expression profiles were shown in Figures 6G,H. Collectively, these results demonstrated that this risk signature could identify high-risk ovarian cancer patients with poor prognosis. Moreover, we confirm the prognostic role of the four-gene risk signature in an independent ovarian cancer dataset in UCSC database (Supplementary Figure 3). Collectively, our results demonstrated that the m^6^A regulators contributed to the progression and prognosis of ovarian cancer.

## Discussioin

Recent studies have demonstrated that m^6^A RNA methylation was implicated in tumorigenesis of various cancers (Esteve-Puig et al., 2020). Previously we explored the role of the specific m^6^A regulator YTHDF1 in ovarian cancer progression and found that YTHDF1 as the m^6^A “reader” could promote protein synthesis in an m^6^A -dependent manner, indicating that m^6^A RNA methylation might have a key value of prognostic prediction for ovarian cancer patients (Liu et al., 2020). In this study, we analyzed genetic variations and gene expression of the 20 m^6^A methylation regulators in TCGA ovarian cancer cohort as well as GSE ovarian cancer datasets. These m^6^A RNA methylation regulators exhibited the low frequency of mutations but the high frequency of copy number variation, which is consistent with previous notion that high grade serous ovarian cancer is driven by genomic copy number changes rather than point mutations (Cancer Genome Atlas Research Network, 2011). Despite that *TP53* was highly mutated in ovarian cancer according to genomic analysis, few genes other than *TP53* are mutated at a high level. Extensive copy number change in more than half of ovarian cancer contributed to tumorigenesis of ovarian cancer (Cancer Genome Atlas Research Network, 2011). In our study, we found that multiple m^6^A RNA methylation regulator genes were amplified in ovarian cancer. Especially, the m^6^A “reader” IGF2BP2 gene was predominantly amplified with a frequency of 18% in ovarian cancer and the high amplification rate of IGF2BP2 was also reported in other cancers. IGF2BP2 was involved in the development of various cancers including colorectal carcinoma, liver cancer and cervical cancer through recognizing m^6^A modified RNAs and regulating RNA stability and translation. Further investigations are warranted to confirm the role of IGF2BP2-mediated fate regulation of m^6^A modified RNA in ovarian cancer. Besides gene amplification of multiple m^6^A RNA methylation regulators in ovarian cancer, most of the m^6^A RNA methylation regulators exhibited upregulation at the RNA level in ovarian cancer such as METTL3, KIAA1429, HNRNPC, ZC3H13 as well as IGF2BP2, suggesting the critical unexplored functions of m^6^A RNA methylation in ovarian cancer.

Notably, the m^6^A writers METTL3, KIAA1429, METTL14, and WTAP constructed the hub genes in m^6^A RNA methylation regulator interaction network according to PPI in ovarian cancer. Though acting as m^6^A writers, these m^6^A RNA methylation regulators exerted distinct effects on the progression of different cancers. For instance, METTL3 initiated m^6^A mRNA methylation to promote drug resistance and metastasis of non-small-cell lung cancer by enhancing the translation and activity of YAP (Jin et al., 2019). On the contrary, reduced METTL3 expression followed by reductions in m^6^A methylation increased AKT activity and thus promoted the proliferation and tumorigenesis of endometrial cancer (Liu et al., 2018). Upregulation of METTL14 induced PERP elevation and promoted the growth and metastasis of pancreatic cancer (Wang et al., 2020). However, METTL14 mediated the N6-methyladenosine modification of SOX4 mRNA and suppressed the metastasis of colorectal cancer (Chen et al., 2020). In terms of WTAP, it acted as an oncogene in hepatocellular carcinoma and high-grade serous ovarian carcinoma (Yu et al., 2019). KIAA1429 contributed to the progression of liver cancer and breast cancer (Lan et al., 2019; Qian et al., 2019). These studies suggest that regulatory network formed by m^6^A RNA methylation is complex and depends on cellular contexts. Thus we constructed an m^6^A RNA methylation regulators-based signature for predicting the prognosis of ovarian cancer. According to the four- m^6^A RNA methylation regulator signature, the ovarian cancer patients in both training set and test set could be stratified into high-risk group and low-risk group, and patients in high-risk group had a worse prognosis than that in low-risk group, suggesting its good performance for prognostic prediction. The signature genes included IGF2BP1, KIAA1429, HNRNPA2B1, and ELAVL1, among which HNRNPA2B1 acts as a protective gene. Although our previous study demonstrated that loss of HNRNPA2B1 inhibited the growth and metastasis of ovarian cancer, this oncogenic role of HNRNPA2B1 is likely independent of m^6^A RNA methylation (Yang et al., 2020).

By an unsupervised clustering based on 20 m^6^A RNA methylation regulators, patents in TCGA ovarian cancer cohort were divided into four clusters and different clusters showed the distinct m^6^A RNA methylation patterns and overall survival. Intriguingly, we found higher TME immune cell infiltration as well as higher stroma score in clusters with worse prognostic patients, suggesting that immune cells might be retained in the stroma and were suppressive in these clusters of ovarian cancer patients as the previous study reported (Chen and Mellman, 2017). Subsequently, GSVA enrichment analysis was conducted to comprehensively understand the characterization in clusters with different m^6^A RNA methylation patterns. The results showed that each cluster enriched distinct patterns of key genes and regulatory pathways.

In conclusion, our study explored genetic variation and the prognostic value of m^6^A RNA methylation regulators in ovarian cancer, and a four-gene signature was found to predict the prognosis of ovarian cancer. We also demonstrated the key regulatory pathways associated with m^6^A RNA methylation and more investigation might be required to decode the precise role of specific m^6^A RNA methylation regulators as well as their related genes or regulatory pathways.

## Data Availability Statement

The original contributions presented in the study are included in the article/Supplementary Material, further inquiries can be directed to the corresponding author/s.

## Ethics Statement

In accordance with the local legislation and institutional requirements, ethical review and approval was not required for the study on human participants.

## Author Contributions

PY and TL designed the research study. QW, DY, XL, HZ, YY, and JX analyzed the data. QW, DY, and XL wrote the manuscript and interpreted the data. All authors read and approved the final manuscript.

## Conflict of Interest

The authors declare that the research was conducted in the absence of any commercial or financial relationships that could be construed as a potential conflict of interest.

## Footnotes

1 https://portal.gdc.cancer.gov

2 https://string-db.org

3 http://cibersort.stanford.edu/

4 http://kmplot.com/analysis/index.php?p=background
