# Supplementary material for: Exploration of the Role of m6 A RNA Methylation Regulators in Malignant Progression and Clinical Prognosis of Ovarian Cancer
Source: Front Genet. 2021 Jun 3;12:650554. doi: 10.3389/fgene.2021.650554 (PMC8209520; doi:10.3389/fgene.2021.650554)
Supplement: Supplementary file 1 [file Data_Sheet_1.docx]

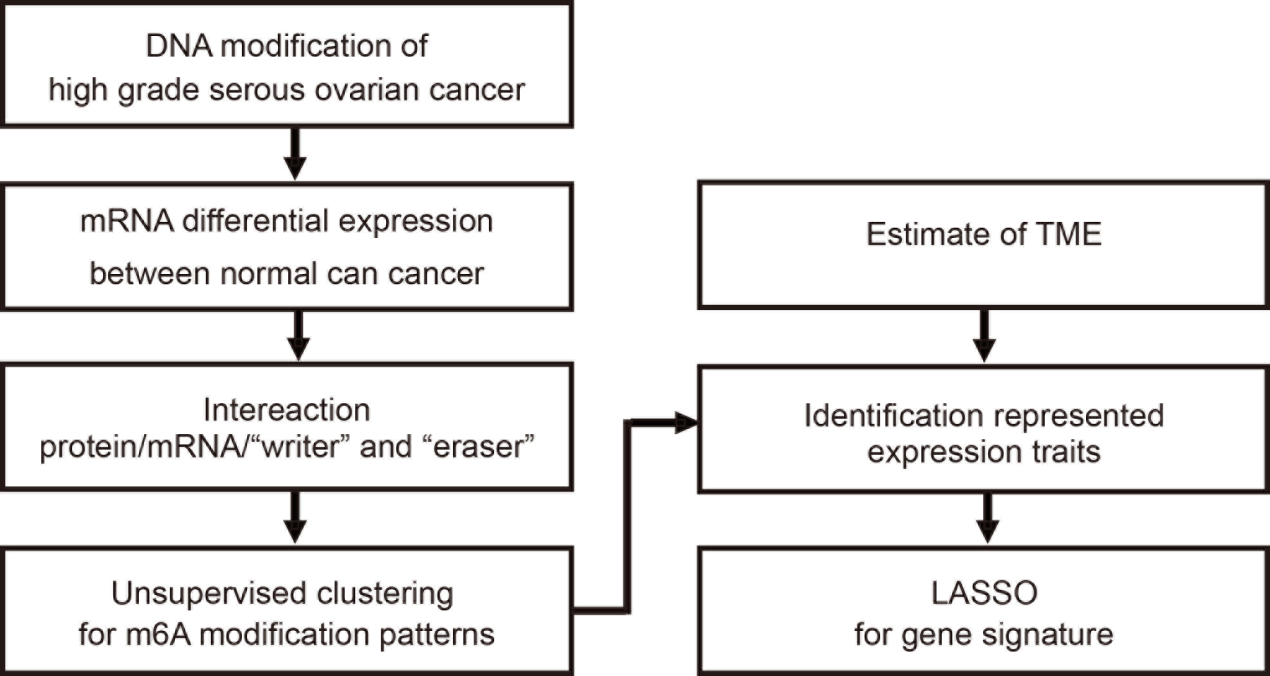
**Figure S1.** Overview of this work


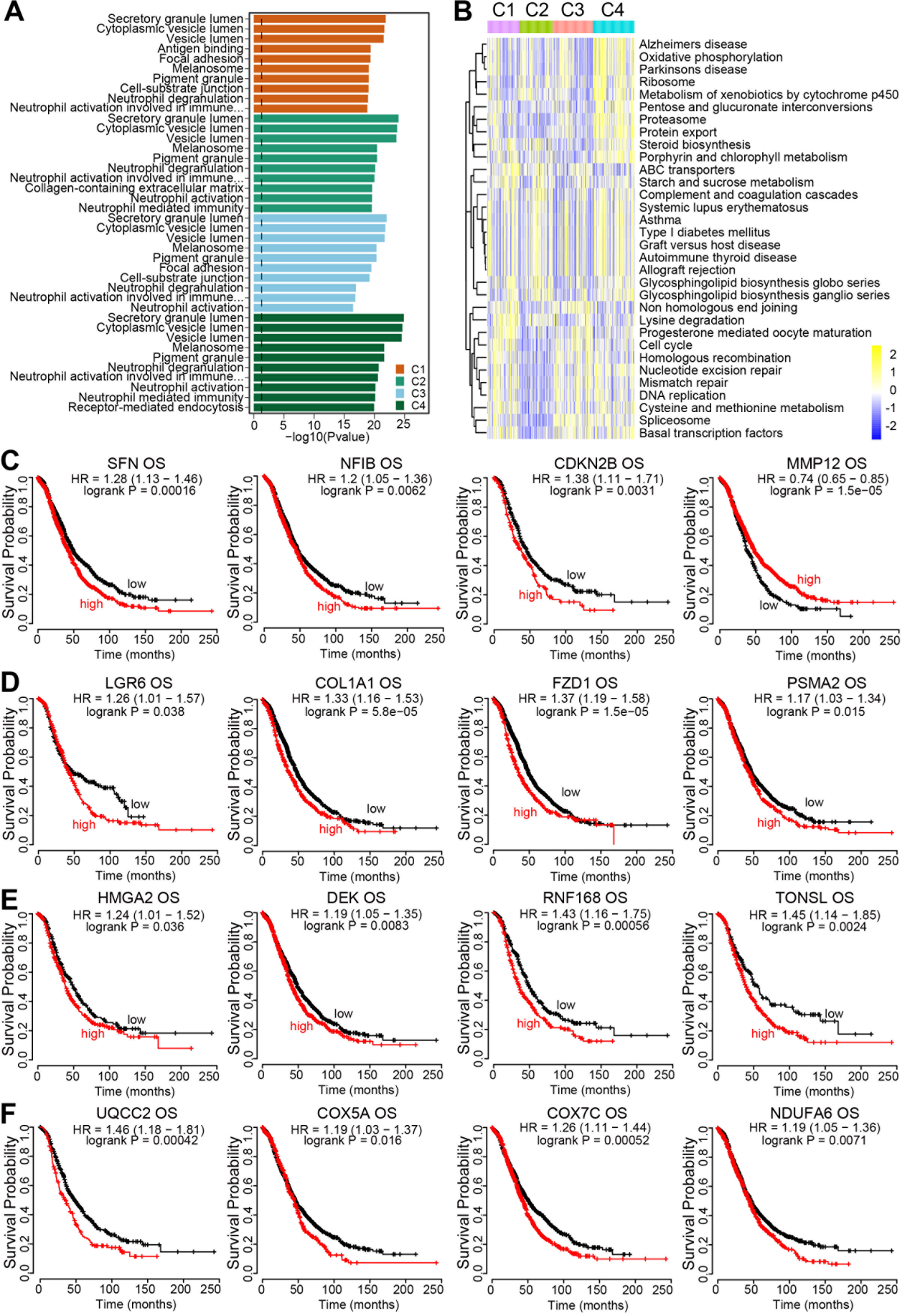


**Figure S2.** **(A)** The most enriched 10 GO terms using the top 500 expressed genes in each m^6^A modification patterns. **(B)** GSVA analysis of each m^6^A clusters. **(C-F)** Overall survival curves of representative genes in each m^6^A modification patterns.

**
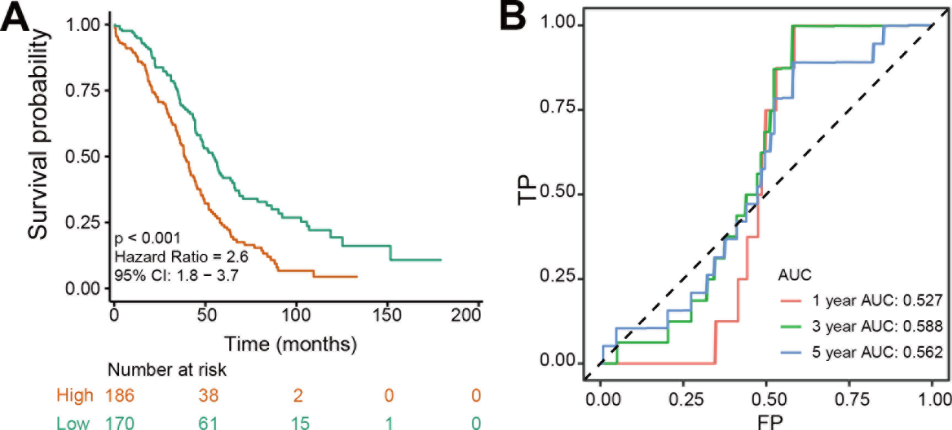
**

**Figure S3. (A)** The survival analysis of UCSC ovarian cancer dataset which was divided by the cutoff value according risk score calculated by LASSO multivariate. **(B)** The ROC curve for evaluating the prediction efficiency of the prognostic signature.
